# Missed Diagnosis of Aortic Intramural Hematoma Presenting With Hemorrhagic Pericardial Effusion: A Case Report

**DOI:** 10.1155/cric/1639392

**Published:** 2025-11-03

**Authors:** Borche Pavlov, Stephanie Döll, Carmen Burca-Arvatu, Georg Dubslaff, Philip Lauten

**Affiliations:** ^1^Department of Cardiology and Internal Intensive Care Medicine, Zentralklinik Bad Berka GmbH, Bad Berka, Thuringia, Germany; ^2^Interdisciplinary Emergency Center, Zentralklinik Bad Berka GmbH, Bad Berka, Thuringia, Germany; ^3^Center for Anesthesia, Intensive, and Emergency Medicine, Zentralklinik Bad Berka GmbH, Bad Berka, Thuringia, Germany; ^4^Center for Diagnostic/Interventional Radiology and Neuroradiology, Zentralklinik Bad Berka GmbH, Bad Berka, Thuringia, Germany

**Keywords:** acute aortic syndrome, intramural hematoma, missed diagnosis, pericardial effusion, transesophageal echocardiography

## Abstract

Acute aortic syndrome (AAS), encompassing aortic dissection, aortic intramural hematoma (AIH), and penetrating aortic ulcer, is a life-threatening condition requiring prompt diagnosis and often immediate surgical intervention. A 65-year-old woman presented with transient loss of consciousness and hemorrhagic pericardial effusion. Initial imaging revealed no clear aortic pathology, leading to diagnostic uncertainty despite clinical suspicion of AAS. Following pericardiocentesis, her condition temporarily stabilized but later deteriorated, revealing a missed diagnosis of aortic dissection with tamponade. Retrospective analysis of initial imaging identified subtle aortic wall thickening, indicative of AIH, overlooked at two hospitals.

This case highlights the diagnostic challenges in atypical presentations of AAS and emphasizes the need for comprehensive imaging protocols, careful interpretation of ancillary findings, and repeat evaluations to prevent misdiagnosis. Clinicians should maintain a high index of suspicion for acute aortic pathologies in patients with hemorrhagic pericardial effusion, ensuring timely and accurate diagnosis to improve outcomes.

## 1. Introduction

Acute aortic syndromes represent a spectrum of life-threatening conditions involving the aorta, including aortic dissection (AD), aortic intramural hematoma (AIH), and penetrating aortic ulcer (PAU). AIH, the focus of this case, is a particularly challenging diagnosis due to its subtle presentation and potential for rapid progression. Although AIH can initially mimic other cardiovascular emergencies, it is unique in its pathophysiology, marked by the development of hematoma within the aortic wall without a visible intimal tear. This feature makes prompt and accurate diagnosis crucial, as delayed or missed recognition can lead to devastating complications, including dissection and rupture.

In Western populations, AIH is relatively rare, comprising approximately 6% of acute aortic syndromes. Diagnosing AIH requires high clinical suspicion and careful imaging interpretation, as findings may be subtle and can vary with different imaging modalities. ECG-gated computed tomography (CT) and transesophageal echocardiography (TOE) are essential tools, though even these can occasionally miss critical features if not interpreted in the correct clinical context (Table 1).

This case report details the presentation, imaging, and management challenges of a 65-year-old woman with AIH, illustrating both the complexity of diagnosing AIH and the life-saving importance of timely intervention. It highlights the nuances in imaging interpretations that are vital to distinguishing AIH from other aortic pathologies and underscores the need for careful monitoring and prompt treatment in cases presenting with hemorrhagic pericardial effusion and suspected acute aortic syndrome.

## 2. Case Presentation

A 65-year-old woman experienced sudden sharp pain between her shoulder blades while driving, causing her to stop and briefly lose consciousness. Emergency services arrived to find her partially conscious. After regaining full awareness, she reported only nausea. She was taken to an external clinic's emergency department.

Her clinical exam was unremarkable, but her D-dimer levels were elevated at 3.6 mg/L (reference 0–0.32 mg/L). A non-ECG-triggered chest CT to rule out pulmonary embolism revealed a large hemorrhagic pericardial effusion and aortic ectasia. Suspecting acute aortic syndrome, she was transferred to our hospital for further evaluation.

**Figure 1:**
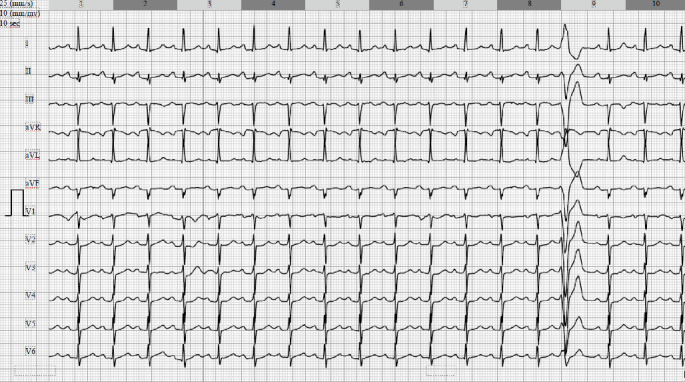
Baseline electrocardiogram showed sinus tachycardia at 108 bpm with borderline low voltage in the precordial leads.

Upon arrival, she was generally stable but intermittently hypotensive, managed with catecholamines. Her ECG showed sinus tachycardia (Figure 1), and a transthoracic echocardiogram (TTE) confirmed significant pericardial effusion. Due to the findings of aortic ectasia and hemorrhagic pericardial effusion, which could suggest an acute aortic syndrome and potentially justify surgical intervention, but without clear evidence of acute aortic pathology, a repeat contrast-enhanced, ECG-gated CT scan of the aorta was performed to specifically assess for AD. This scan again ruled out dissection.

Consequently, the patient was admitted to the cardiology unit with a working diagnosis of hemorrhagic pericardial effusion of unknown etiology, with a recommendation for further differential diagnostic evaluation. The following day, pericardiocentesis was performed, removing approximately 500 mL of bloody effusion, and a drainage catheter was placed. She returned to the monitoring unit in stable condition, with a follow-up echocardiogram showing no significant residual effusion.

**Figure 2:**
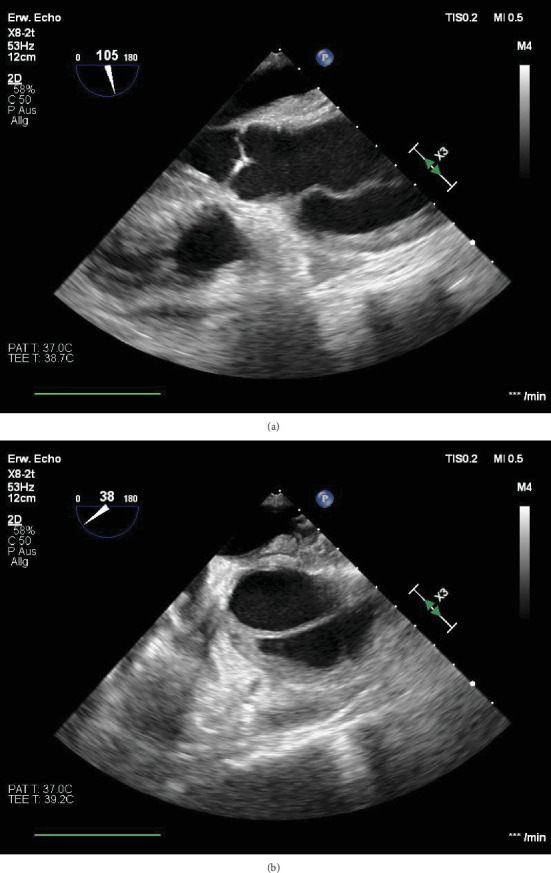
(a) Preoperative transesophageal echocardiography (TOE) of the aorta (longitudinal axis), showing distinct aortic wall thickening and the presence of a dissection membrane. (b) Preoperative transesophageal echocardiography (TOE) of the aorta (short axis), showing the same wall thickening and the dissection membrane.

Approximately 48 h after the initial presentation, the patient developed pulseless electrical activity, requiring resuscitation. Suspecting retamponade, an urgent exploratory thoracotomy was planned; however, preoperative TOE revealed an AD (Figure 2a,b and Videos S1 and S2). After transfer to the operating room, the patient was placed on cardiopulmonary bypass. Intraoperative findings revealed an extensive aortic wall hematoma and multiple tears, prompting replacement of the ascending aorta and complete arch with vascular grafts, followed by decannulation. Unfortunately, minutes later, the patient experienced cardiac arrest and died on the operating table.

**Figure 3:**
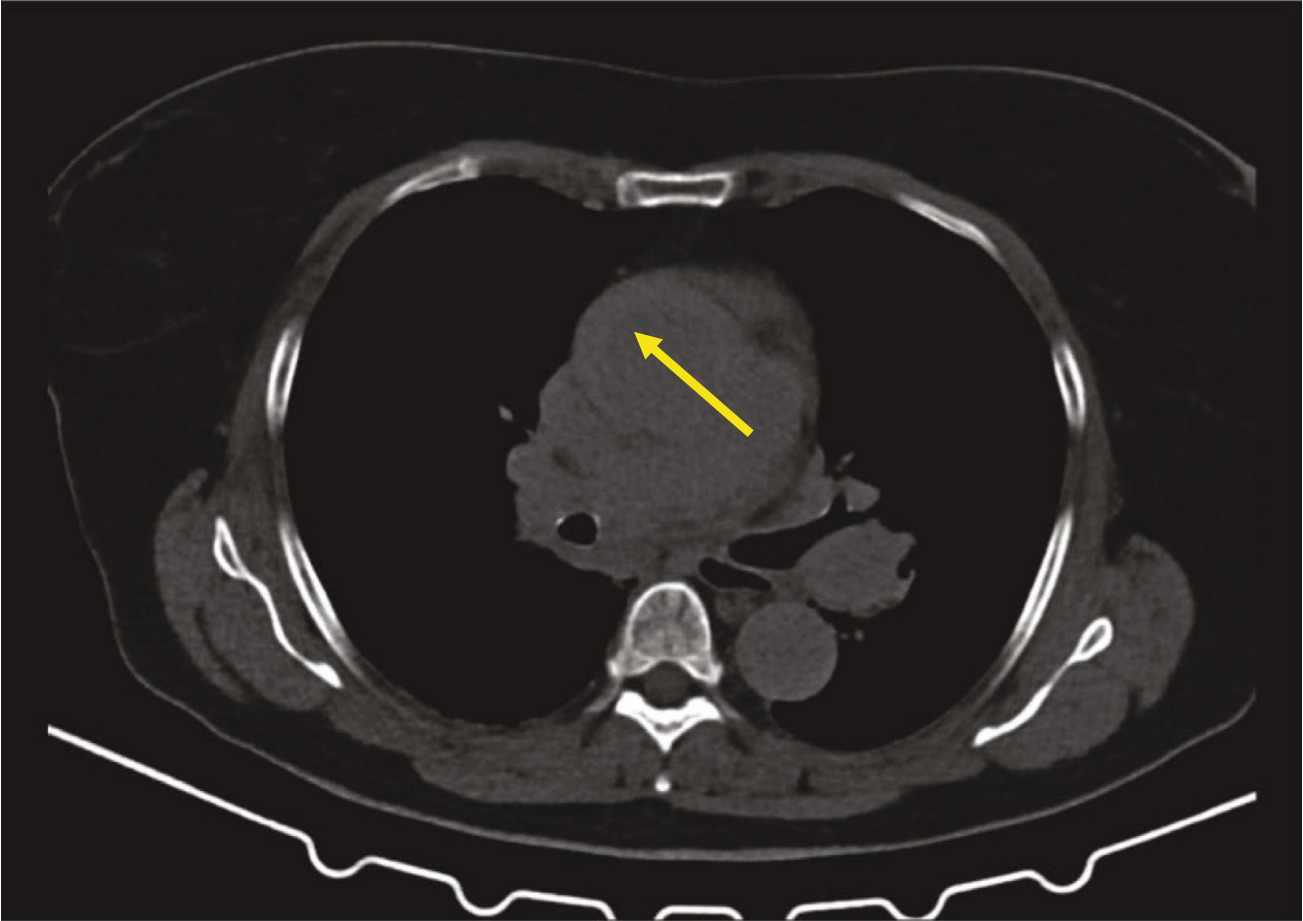
Plain computed tomography of the aorta performed at the referral hospital showed a focal, crescent-shaped, high-attenuation region with eccentric thickening of the aortic wall (indicated by an arrow), with a total diameter of approximately 48 mm.

**Figure 4:**
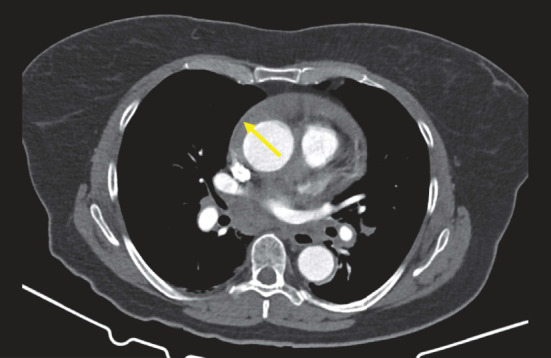
Contrast-enhanced computed tomography of the aorta, performed at the referral hospital on a slice corresponding to Figure 3, shows a contrast-filled aorta without visible flaps and with a normal diameter. However, a subtle delineation between the aortic wall hematoma and the pericardial effusion remains visible (indicated by an arrow).

Postmortem, we thoroughly reviewed all CT data from both thoracic scans for signs of acute aortic syndrome. In the scans performed at the referring hospital, thickening of the aortic wall consistent with an AIH was identified in both the noncontrast and contrast-enhanced non-ECG-gated thoracic CT scans, clearly distinguishable from the pericardial effusion; however, these findings require substantial radiological experience for detection and were not recognized during the initial interpretation (Figures 3 and 4).

**Figure 5:**
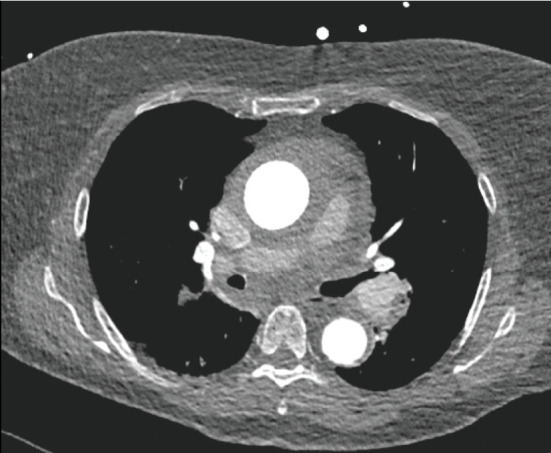
Contrast-enhanced computed tomography of the aorta, performed at our institution, showing a vessel with a normal diameter of 38 mm without visible dissection membrane or aortic wall thickening. The aorta is therefore described as free of pathology.

Additionally, this subtle finding was not clearly visible on ECG-triggered contrast-enhanced CT images of the aorta performed at our institution due to larger slice thickness and contrast density, which likely contributed to it being missed during interpretation (Figure 5).

## 3. Discussion

Acute aortic syndromes are life-threatening emergency conditions characterized by similar clinical features involving the aorta. They are traditionally classified into three categories: AD, PAU, and AIH [1].

An AIH forms when a hematoma develops within the media of the aortic wall, without an entry tear or intimal flap. It is diagnosed when there is circular or crescent-shaped thickening of the aortic wall > 5 mm, as normal thickening is < 3 mm and lacks detectable blood flow. Over time, it can cause a secondary intimal tear or extend into the adventitial space, potentially leading to overt AD or rupture [1, 2].

The pathology is generally thought to result from rupture of the medial vasa vasorum for which hypertension and atherosclerosis play a major role in most patients, causing hemorrhage to spread within the arterial media and subsequent thrombosis, but new insights suggest also alternative mechanisms, such as plaque rupture in aortic wall or sealed tear which cannot be seen on imaging studies, could also play a role [3].

The prevalence of AIH in Western countries, according to IRAD registry data, ranges from 5% to 20%, with an average of 6.3% of all acute aortic syndromes [4]. In contrast, the ASAN AAS registry reports a prevalence of 33.6%, indicating a significantly higher incidence in East Asian populations [5]. It remains unclear whether this difference represents true variation due to differing risk factors, or simply reflects reporting discrepancies, particularly considering the disease's dynamic and progressive nature.

Data from clinical registries show that the clinical presentation and 1-year outcomes of patients with AIH are similar to those with acute AD. However, compared to AD, AIH patients tend to be older, females are more frequently affected, and more patients have cardiovascular risk factors, such as hypertension, which is present in over 90% of AIH cases. The primary symptom, reported by more than 80% of patients, is chest or back pain, while syncope is rare [4–6].

Complications like neurological deficits or limb ischemia are less frequent in AIH than in AD. Cardiac complications, including aortic regurgitation, are rare, although pericardial effusion is common, observed in 60% of AIH patients; up to one-third may develop cardiac tamponade [7]. Following the Stanford classification, AIH can be categorized as Type A (42%) involving the ascending aorta or Type B (58%) involving only the descending aorta.

The D-dimer test is particularly effective in excluding AIH, with a cutoff value of 500 mg/mL yielding 100% sensitivity and 67% specificity, making a negative test highly reliable for ruling out AIH in low-pretest probability patients [8].

TTE is a useful first-line imaging tool for suspected AIH due to its accessibility, although it has low sensitivity for AIH itself. However, it reliably identifies high-risk complications like pericardial effusion. TOE provides superior imaging due to its proximity to the aorta, allowing high sensitivity and specificity in identifying aortic wall thickening and dissection. However, its limitations include invasiveness and sedation requirements [9].

CT is essential for diagnosing, risk-stratifying, and managing acute aortic diseases, with sensitivity and specificity rates approaching 100% in their detection, especially in ADs [10]. ECG-gated acquisition is recommended to minimize motion artifacts near the aortic root, ideally starting with a non-contrast CT followed by contrast-enhanced CT angiography, particularly if AIH is suspected. However, in clinical practice, suspicion of acute aortic syndrome frequently leads directly to contrast-enhanced CT without a preliminary noncontrast scan. Noncontrast CT is critical for detecting the high-attenuation, crescent-shaped thickening of the aortic wall—a hallmark of AIH—that may be missed on contrast-enhanced CT, as seen in our case. Hemorrhagic pericardial effusion, commonly associated with AIH, is easily detected on plain CT when pericardial fluid density exceeds 40 HU [11].

As seen in our case, hemorrhagic pericardial effusion can present a significant diagnostic challenge, as it may arise not only from acute aortic pathology but also from broader conditions such as neoplastic or infectious pericarditis. Differentiation requires integration of clinical presentation, laboratory findings, and advanced imaging—particularly CT and cardiac MRI—to distinguish between these entities. In particular, patients with unexplained hemorrhagic effusion and acute illness should raise suspicion for an AIH, as this association is well recognized [12].

Rare causes mimicking acute aortic pathology with hemodynamic compromise have also been reported. For example, Cava et al. described a spontaneous aortic valve laceration presenting with cardiac tamponade and sudden severe aortic regurgitation, requiring urgent surgical repair [13]. This highlights how uncommon pathologies can mimic typical acute aortic syndromes and underscores the importance of a broad differential diagnosis in patients with pericardial effusion and hemodynamic instability.

Hemorrhagic pericardial effusion in the context of AIH can be one of the signs indicating progression to acute AD, which can occur in up to one-third of patients [14, 15]. This necessitates careful evaluation and consideration of emergency surgery, especially if high-risk features such as persistent pain, hemorrhagic effusion, or aneurysmal dilation of the aorta are present [15].

## Figures and Tables

**Figure 6:**
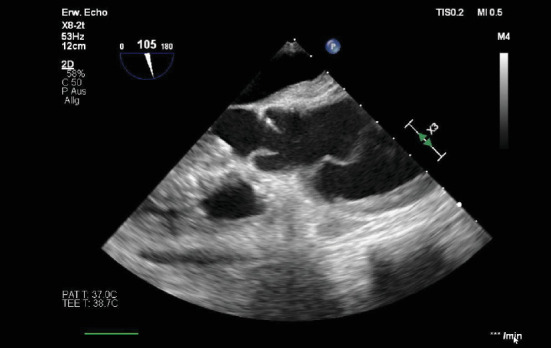
Preoperative transesophageal echocardiography (TOE) of the aorta in the longitudinal axis, with a visible dissection membrane and mural hematoma.

**Figure 7:**
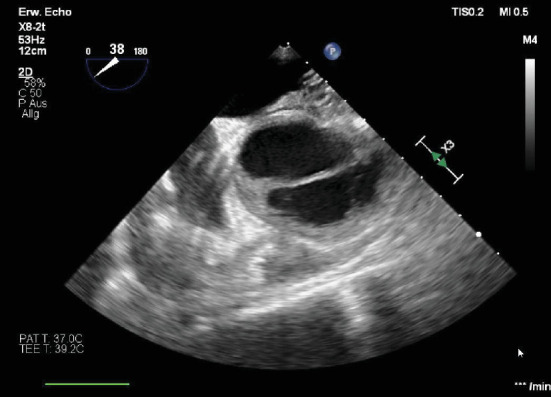
Preoperative transesophageal echocardiography (TOE) of the aorta in the short axis, with a visible dissection membrane and mural hematoma.

**Table 1:** Clinical timeline.

**Time (hours)**	**Event**
0 h	Sudden back pain, brief loss of consciousness while driving
1 h	Arrival at external hospital; non-ECG-gated CT → hemorrhagic pericardial effusion detected
3 h	Transfer to our center for further evaluation
4 h	ECG-gated CT → no dissection visible; planned diagnostic and therapeutic pericardiocentesis; patient transferred to ICU
24 h	Pericardiocentesis performed (500 mL blood removed); patient stabilized in ICU
48 h	Clinical deterioration; pulseless electrical activity → TOE reveals AIH/dissection
50 h	Emergency surgery; extensive aortic replacement performed
50+ h	Patient suffers intraoperative cardiac arrest and dies

## Data Availability

The data that support the findings of this study are available from the corresponding author upon reasonable request.

## Supporting Information

Supporting Information 1 Video S1 showing the preoperative transesophageal echocardiography (TOE) of the aorta (longitudinal axis) with a dissection membrane.

Supporting Information 2 Video S2 showing the preoperative transesophageal echocardiography (TOE) of the aorta (short axis) with a dissection membrane.
